# Cross-Reactivity of TCR Repertoire: Current Concepts, Challenges, and Implication for Allotransplantation

**DOI:** 10.3389/fimmu.2016.00089

**Published:** 2016-03-24

**Authors:** Nicolas Degauque, Sophie Brouard, Jean-Paul Soulillou

**Affiliations:** ^1^UMR 1064, INSERM, Nantes, France; ^2^ITUN, CHU de Nantes, Nantes, France; ^3^Faculté de Médecine, Université de Nantes, Nantes, France; ^4^LabEx IGO “Immunotherapy Graft Oncology”, Nantes, France; ^5^CIC Biothérapie, Nantes, France; ^6^CRB, CHU Nantes, Nantes, France; ^7^LabEx Transplantex, Nantes, France

**Keywords:** TCR repertoire, transplantation, cross-reactivity, alloreactivity, TCR, MHC, T cell

## Abstract

Being able to track donor reactive T cells during the course of organ transplantation is a key to improve the graft survival, to prevent graft dysfunction, and to adapt the immunosuppressive regimen. The attempts of transplant immunologists have been for long hampered by the large size of the alloreactive T cell repertoire. Understanding how self-TCR can interact with allogeneic MHC is a key to critically appraise the different assays available to analyze the TCR Vβ repertoire usage. In this report, we will review conceptually and experimentally the process of cross-reactivity. We will then highlight what can be learned from allotransplantation, a situation of artificial cross-reactivity. Finally, the low- and high-resolution techniques to characterize the TCR Vβ repertoire usage in transplantation will be critically discussed.

## Understanding the Cross-Reactivity

### Shaping the T Lymphocyte Receptor Repertoire

**Figure 1:**
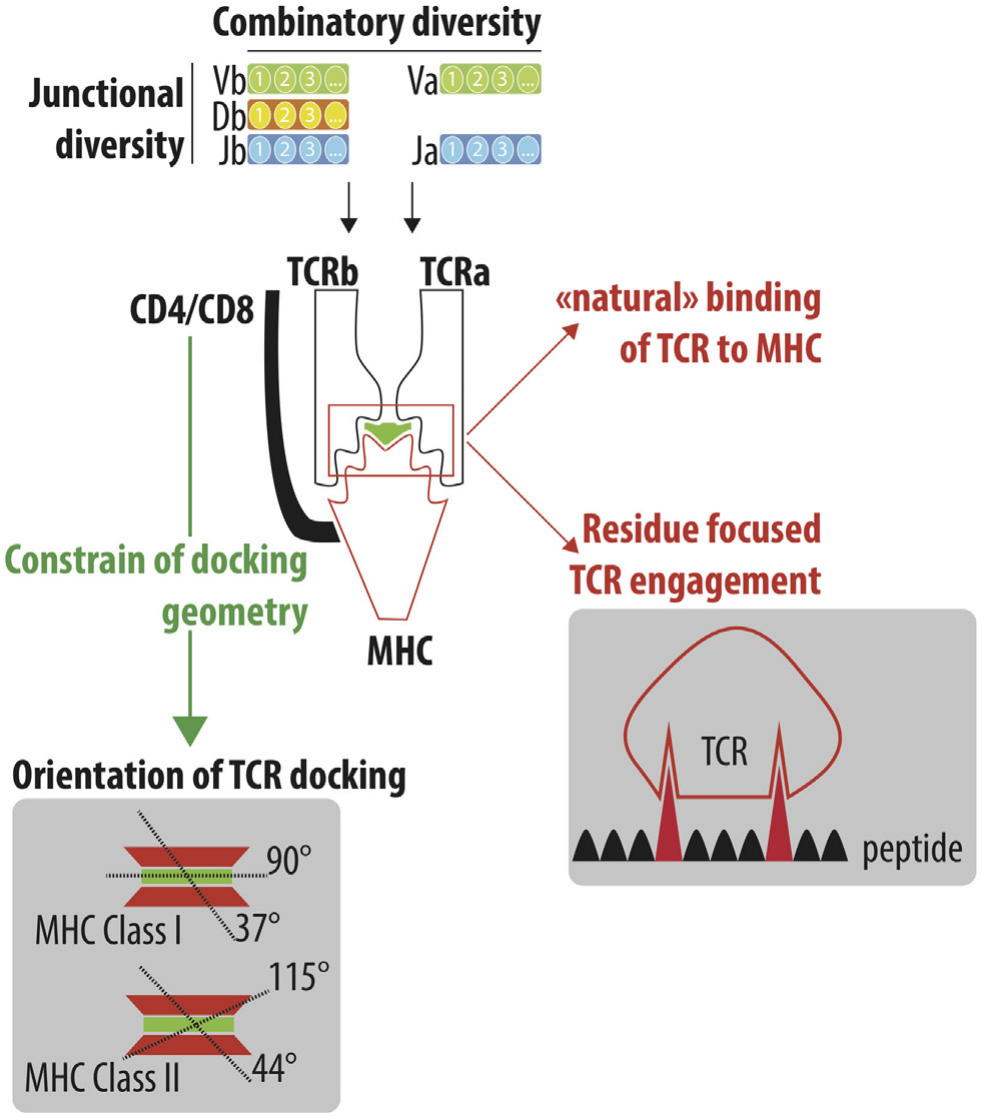
**Understand the cross-reactivity of a highly diverse TCR repertoire**. A highly diverse TCRαβ repertoire is generated by iterative processes selected through evolution. Combinatory diversity results from the selection of Variable (V; TRAV and TRBV), Diversity (D; TRBD) and Joining (J; TRAJ and TRBJ) genes. Junctional diversification further extends the combinatorial diversity by either trimming gene ends or adding nucleotides between the recombining genes. Finally, the association of the TCRα and TCRβ chain constitutes the final steps of the numerous iteration processes that lead to the generation of a highly diverse TCR repertoire, which is able to efficiently protect individuals from pathogenic stimulations. TCRαβ adopts a stereotype docking geometry atop the MHC/peptide complex. This orientation leads to a spatial interaction between the germline-encoded CDR1 and CDR2 of the TCRα and β chains and the edges of the peptide-groove of MHC. The accumulation of reported crystallographic structures has challenged the stereotypic view of the angle of the TCR docking. However, the recognition of conserved motifs on the side of MHC molecules by CD4/CD8 co-receptor constrained the TCR docking geometry. Despite the high diversity of the TCR repertoire, a high degree of cross-reactivity has been reported that could be explained by the “natural” ability of TCR to interact with MHC molecules (MHC focus model) as well as the interaction of TCR to a limited number of amino acids of the peptide bound to the MHC peptide groove.

Through evolution, numerous processes have been selected to generate a diverse repertoire of TCRαβ able to protect mammalian from pathogenic insults (Figure 1). Highly similar genes recombine to form functional genes and generate a highly diverse TCR repertoire. TCRβ chains are encoded by distinct Variable (V; TRBV), Diversity (D; TRBD), and Joining (J; TRBJ) genes, whereas TCRα chains are encoded by distinct sets of V and J genes (TRAV and TRAJ). Junctional diversification further extends the combinatorial diversity by either trimming gene ends or adding nucleotides between the recombining genes (1). In contrast to the IGHV (V genes of Immunoglobulin Heavy Chain) germline dataset compiled by the ImMunoGeneTics (IMGT) group that greatly benefit from the advanced of deep-sequencing technologies, the human TCR germline has been only minimally changed since the complete sequencing of the TCR gene loci in 1996 (2, 3). The 65 functional genes, ORFs, and pseudogenes have been reported for the TRBV, 54 for the TRAV and 2 for the TRBD dataset. The analysis of the TCR CDR3 is still a very challenging process. The identification of the TRBD genes cannot be performed due to the high degree of similarities of the TRBD at their 5′ ends, the short length of the two genes, and the presence of G-rich N nucleotides at the 5′ ends that could be also added by the TdT enzyme.

It is misleading to estimate the combinatory diversity by simply multiplying together the number of V, D, and J genes (4). Rather than a random combination of the TCR genes, studies have shown that TCR genes are highly biased in their usage, and that only part of the theoretical diversity is selected (5, 6). Chromosomal recombination patterns can be explained by variations in enhancers and Recombination Signal Sequences (RSS) and organization of the TRBJ genes (a block of six and seven genes located respectively downstream from the TRDB1 and TRDB2 gene) that leads to a bias in D–J pairing. The diversity of the TCR repertoire is further broaden during the rearrangement process first by the addition of P nucleotides (Palindromic nucleotides) thanks to recombination activating gene-1 and -2 (RAG1 and RAG2) (7) that form hairpin loops at the gene end and then by the addition of N nucleotides (with a biased toward G nucleotides) by the terminal deoxynucleotidyl transferase (TdT) (8). Insertions of nucleotides have a profound impact on the diversity of the Complementary-Determining Regions 3 (CDR3) sequences and contribute to most (60%) of this diversity (9). The coding ends of the genes can be also trimmed by exonucleases. However, given the limited number of amino acids, the removal of nucleotides by exonucleases is constrained to generate a productive codon and therefore limits the contribution of exonuclease trimming to the diversity of the TCR repertoire. Finally, the association of the TCRα and TCRβ chain constitutes the final steps of the numerous iteration processes that lead to the generation of a highly diverse TCR repertoire, which is able to efficiently protect individuals from pathogenic stimulations.

### Current Understanding of the Recognition of pMHC by TCR

Six CDR will engage the peptide/MHC complexes, endogenous and exogenous peptides being presented respectively by MHC class I and II molecules. MHC class I grooves constrain the length of the presented peptides (8–14 amino acids length) while the open nature of peptide-binding cleft of MHC class II molecules allow a broader range of peptides to be presented. The HLA locus is the most polymorphic region of the human genome, with more than 13,000 variant alleles (10,297 HLA Class I Alleles and 3,543 HLA Class II Alleles according to the IMGT/HLA). The high diversity of HLA conferring an almost unique signature of HLA for mankind is further extended by the combinatory diversity resulting from the association of six HLA Class I (two alleles of HLA-A, -B, and Cw) and six HLA Class II molecules (two alleles of HLA-DR, -DP, and -DQ). The high mutation level of the HLA loci is preferentially focused on the peptide-binding cleft that clustered most of the variability of the amino acid sequence. The focus of mutations underlines the function of the HLA molecules, namely being able to display a very large array of peptides.

Garcia et al. were the first to report the crystallographic structure of a murine TCR 2C bound to peptide/MHC Class I (H-2K^B^–dEV8). The cytotoxic T cell clone 2C is one of the most well-characterized TCR and has been initially isolated from a BALB/b mouse as an allospecific T cell that recognized L^d^ on the mastocytoma P815. Beside its primary antigen (peptide p2C), the 2C TCR can bind to different antigens, including the dEV8 (10) and SIYR (11). They also showed that TCRαβ adopts a 45° diagonal orientation to the long axis of the peptide (12). This orientation leads to a spatial interaction between the germline-encoded CDR1 and CDR2 of the TCRα and β chains and the edges of the peptide-groove of MHC (Figure 1). The highly diverse CDR3 region is facing the central portion of the bound peptide. The multiple crystallographic structures of TCR/peptide MHC complexes [more than 120 of crystallographic structures have been obtained (13)] have revealed that the docking angle of the TCR is conserved with a stereotype position of a 75° diagonal orientation to the long axis of the peptide (14). The conserved binding model has lead to the concept that TCR and MHC are hardwired to interact, resulting from a coevolution selection of conserved regions (codons) to lock in TCR onto MHC molecule. The stereotyped orientation of TCR atop MHC molecule is however more flexible than initially proposed, with the accumulation of crystal structures. The median docking angle of TCR is 63.2° (min–max 37–90°) with MHC class I and 76.4° (min–max 44–115°) with MHC class II (13) (Figure 1).

Different theories have been postulated to explain the hardwire of TCR to MHC molecules (15), including the key role of co-receptors of CD4 and CD8 that imposed steric requirements for concurrent associations of TCR, CD3, CD4/CD8, and MHC complexes allowing the appropriate signaling events to occur. Indeed, the main role of co-receptor CD4 and CD8 is to recruit the Src tyrosine kinase p65lck (lck) via its association with the cytoplasmic tail of CD4 or CD8. Lck concentration promotes phosphorylation of immunoreceptor tyrosine activation motifs (ITAMs) in the cytoplasmic tails of CD3 subunits and then initiates the cascade of signaling events leading to the full activation of the T lymphocyte. Given the key role of co-receptor CD4 and CD8 in process, it was assumed that their ability to bind, respectively, the membrane-proximal α2 and β2 domains of the MHC class II molecule and the protruding loop in the α3 domain of the MHC class I molecule will constrain the docking geometry of the TCR to the pMHC (Figure 1).

A recent report by Beringer et al. (16) had challenged the consensus idea of a highly stereotype docking of TCR atop MHC molecules (13). Crystallographic structures of two TCR binding to proinsulin peptide presented by HLA-DR4 (HLA-DR4^proinsulin^) have been obtained from two clones of induced regulatory CD4 T cells. The ternary complexes revealed a 180° polarity reversal compared to all other TCR-peptide-MHC complex structures. It remains to be address whether this singular observation could be generalizable, whether the reverse docking is a unique feature of regulatory cells and whether the potential signaling differences may influence the phenotype and the function of the T cells.

### Cross-Reactivity, from Intellectual Concept to a Critical Need for Immune System

Cross-reactivity can be defined by the ability of a given TCR to interact with more than one pMHC complex with different presented peptides or MHC molecules. This new concept has been presented as early as 1977 by Matzinger and Bevan (17). An alloreactive T cell clone was derived by Owens et al. in 1984 with three H2-E reactivity (allo-E^k^ specific, H2-E^k^, DBA/B10 H2-E^d^, and self H2-E^d^) (18). Since then, numerous reports have provided evidence of cross-reactivity. For instance, mouse 2C TCR can interact with syngeneic MHC H-2K^b^ presenting dEV8 (10) and SIYR (11) and with allogeneic H-2K^bm3^ presenting dEV8/K^bm3^ (10) and allogeneic H-2L^d^–p2CA (11). The study by Birnbaum et al. is an elegant attempt to quantify the cross-reactivity of a given TCR (19). Using five different CD4 TCR clones (three from mouse origin and two from human origin), high throughput screening of yeast libraries and deep sequencing, the authors demonstrate that a single TCR can interact with more than 100 different peptides.

Jerne et al. postulated in the mid-1950 that each cell exhibits a unique clonotype able to recognize only one antigen (20, 21). Don Mason has been among the first to challenge the validity of this clonal selection theory (22) showing that the immune system will be highly incompetent to protect an individual from external insult if one and only TCR was able to recognize a single peptide presented in a given HLA context. More than 10^15^ T cells, which would weigh more than 500 kg, would be needed to provide efficient coverage of the potential foreign peptides. This clearly stated that the immune system could not efficiently protect individual if one TCR interacts with a single antigen. Unlike the affinity maturation of B cell receptor, the protein sequence of TCR is fixed and naive T cells are required to recognize foreign antigens not encountered before. The number of potential antigens to be recognized is huge given the variability induced by the high diversity of peptide-binding groove of HLA class I and II molecules. From the 20 proteinogenic amino acids and given that peptides from 8- to 14-mer can be presented, an incredibly high number of peptides can be potentially generated (>10^15^ peptides) (23). The diversity can be further extending by the posttranslational modifications of amino acids. In a 2012 opinion paper, Andrew Sewell elegantly presents the necessity of the cross-reactivity (23), as the number of potential foreign peptide–MHC complexes that T cells might encounter dwarfs the number of TCRs available [the number of unique TCRαβ is estimated to be in the magnitude of 10^11^ (24, 25)].

The mechanisms described previously to generate a diverse TCRαβ have to be envisioned at the population level. Given the relatively limited number of genes encoding for TCR chain α and β and the requirement of TCR to recognize the highly diverse HLA molecules, the necessity of each T cell to recognize a large array of peptides is expected (22). Before presenting the experimental approach aiming to quantify the number of peptides recognized by a single TCR, we would like to present clear evidences of the cross-reactivity involving memory T cells without previous antigen encounter. It has been described few years ago that CD8 T cells with a memory phenotype can be found in mice (26–28). CD8 T cells specific for ovalbumin and viral antigens (HSV, vaccinia) could be detected in mice despite their germ-free environment (28). Despite the absence of previous antigen encounter, these pre-existing memory CD8 T cells harbor traits of memory cells such as the ability to rapidly proliferate upon stimulation and to secrete rapidly pro-inflammatory cytokines. Homeostatic proliferation, aging, and cross-recognition of alternate ligands have been postulated to drive the accumulation of these memory-like naive CD8 T cells (27, 29). This observation has been extended to human settings in which CD4 T cells specific for HIV-1, CMV, and herpes simplex virus (HSV) epitopes were identified in healthy volunteers that had never been infected with these viruses (30). Again, these cells exhibit not only memory markers but also memory-associated features (rapid proliferation and cytokine secretion). The acquisition of memory characteristics could be a consequence of homeostatic proliferation (31) or a consequence of the cross-reactivity to other antigens in the environment. To support the latest hypothesis, Su et al. have shown that HIV-1 specific T cells can recognize environmental peptides present in the gut and soil, bacteria and ocean algae, and plants. Of interest, T cells specific for HIV-1 can even be purified from cord-blood (30), demonstrating thereby the presence of T cells able to recognize self and non-self antigens in newborns. Of interest, the phenotype of cross-reactive T cells was different between newborns and adults, with a naive and a memory phenotype, respectively.

The concept of clonal deletion that occurred in the thymus is challenged by the aforementioned reports and compelling evidences suggest that from an evolutionary perspective, the necessity to protect an individual against pathogens is far more important than to limit the autoreactivity. A recent study from Davis team further sustained this claim (32). The frequency of CD8 T cells specific of a Y chromosome specific antigen (equivalent to HY peptide) is only threefold lower in man as compared to women (32). Of interest, whereas CD8 T cells purified for their specificity regarding a pool of six self peptides do not proliferate after stimulation with the same set of self peptides, CD8 T cells specific of a pool of six non-self peptides exhibit a potent proliferative response (32). The absence of response reported for self-specific CD8 T cells and not for foreign antigen-specific CD8 T cells has been linked to a different genetic programing as compared to the clones purified from woman, with a lower expression of IL-2R, IL-21R, and Bcl-XL (32). Thus, evolution has favored the absence of hole over autoimmune disease (about 1% of incidence). It may seem awkward that the evolution has favor the escape of anti-self specific T cells from thymic selection over a more stringent deletion of all anti-self T cells. A heavier burden of maintaining tolerance is needed to prevent the development of autoimmune diseases. However, the need to defend the immune system against pathogens, especially during childhood, is far greater than the need to prevent autoimmunity as for population’s survival. By limiting the deletion of self-reactive T cells and thanks to the large cross-reactivity of T cells, the holes in the T cell repertoire that pathogens might take advantage of are constrained.

The analysis of the immune system in monozygotic twins is enlightened in many aspects as such studies allow the dissociation between the inborn and the acquired contributions. The team of Davis has recently showed that the heritability of T and B cells parameters declines very rapidly with age (33). At the age of 40 years, the heritability explained less than 10% of the variation in T and B cell parameters. CMV infection is a protypical example of the influence of non-heritable factor on the whole immune system. Indeed, 58% of all parameters measured in discordant twins were influenced by CMV infection (33). The environment carves the immune system of each single individual, with each past immune response heavily imprinting the (present) immune system.

Since the initial observation that immunity against cowpox protects individual from smallpox (34), numerous examples of cross-reactivity had been reported in mice and in human (35). For instance, infections with BCG, influenza A virus (IAV), lymphocytic choriomeningitis virus (LCMV), and murine cytomegalovirus (MCMV) all confer a level of protective immunity against Vaccinia Virus (36–38). The benefit of cross-reactivity as for pan-virus protection is more difficult to assess for obvious reasons. Nevertheless, the numerous example of a single TCR able to recognize different antigens [BCG and Poxviruses (37); Papillomavirus and Coronavirus (39); Influenza virus and Epstein–Barr virus (40)]. The large cross-reactivity of T cells confers a more efficient protection cover using a limited number of T cells that need to screen an incredibly large array of peptides that can be presented by MHC molecules. Beside the efficient use of limited T cell resource, cross-reactivity confers a spatio-temporal advantage to the immune system to scan any infected cells. Cross-reactivity could also be envisioned as an evolution strategy to limit the immune recognition escape.

## Allotransplantation is Not Only an Example of Artifactual Cross-Reactivity but also Gives Clues Regarding the Global Organization of the Immune System

Recipient immune system can interact with foreign HLA molecules under two very different circumstances: pregnancy and transplantation. Thanks to evolution and adaptation of the maternal immune system to the presence of HLA mismatch fetuses, allorecognition during pregnancy is not harmful and could even be beneficial as for mammalian sexual reproduction. Immunological tolerance toward allogeneic fetus is obtained through a complex network of regulatory mechanisms including the lack of expression of classical MHC class I molecules by the placental trophoblast and the expression of non-classical MHC class I HLA-E and HLA-G. More surprisingly, HLA mismatches have been proposed to be beneficial for pregnancy outcome. In the 1960s, Billington reports that the placenta is larger in H-2 incompatible mouse as compared to compatible fetuses (41). HLA compatible fetuses (i.e., similar to maternal HLA) have been shown to be more prone to be aborted (42). In contrast, recipient immune system will potently eliminate an allogeneic graft in the absence of immunosuppressive therapy.

Despite the absence of thymic central selection (43) of potential graft-recipient T cells by allogeneic MHC motifs regarding their ability to recognize allogeneic potential HLA, a large pool of T cells can be activated by donor HLA molecules either through the direct pathway (i.e., donor HLA presenting donor peptides) or the expected processing of foreign MHC molecules, coined as the “indirect pathway” (i.e., recipient HLA presenting donor peptides) in transplantation immunologist jargon. The direct allorecognition pathway represents a unique example of functional and efficient cross reactivity. Two main hypothesizes have been postulated to explain the basis of alloreactivity, emphasizing the role of either MHC molecule or peptide. The polymorphism between donor and MHC molecules could act as an “innate focus” that leads to the activation of unprimed recipient T cells or the allopeptide could be recognized as foreign antigen while allogeneic and self-MHC molecules exhibit a high degree of similarity (Figure 1).

According to the MHC centric model, the peptide plays only a minor role in the process, and alloreactive TCRs recognize structural determinants on the MHC helices of syngeneic or allogeneic MHC. The bias of TCR to interact with MHC molecules supports this theory. Crystal structures of allo-pMHC complexes such as 2C TCR with allogeneic H-2K^bm3^ presenting dEV8/K^bm3^ (44) or BM3.3 TCR with allogeneic pBM1–H-2K^b^ (45) have shown that alloreactive TCRs interact with allogeneic MHC in a similar fashion as with syngeneic MHC. To further support the role of MHC in alloreactivity, it has been reported that some HLA mismatches between donor and recipient are associated with worse graft survival than others, leading to the notion of taboo mismatches based on shape rather than sequence differences (46). For instance, despite a single amino acid in an HLA Class I antigen, mismatches between HLA-B*4402 and HLA-B*4403 is associated with transplant rejection (47) and acute graft-versus-host disease (48). The peptide repertoire bound to HLA-B*4402 or HLA-B*4403 have been shown to be very similar (49). However, a recent report challenges this observation (50). The single amino acid mismatch induced the presentation of more unique peptides by HLA-B*4403 than HLA-B*4402, consistent with the stronger T cell alloreactivity observed toward HLA-B*4403 compared with HLA-B*4402 (50). This observation supports the notion of a peptide focus TCR allorecognition, in the same line as molecular mimicry.

Allorecognition could also involved cross-reactivity between MHC class I and MHC class II or even xeno MHC (51, 52). In 1986, Schilman et al. reported that CD8 T cell clone could be activated by both MHC class I (H-2D^b^) and MHC class II (I-E^k^) molecules (53). By-directional recognition of T cells between MHC class I and MHC class II have been reported later (54–56). These observations may have important implication in the attempt to minimize HLA mismatches during the process of organ allocation.

### Defining the Magnitude of T Cell Response to Allostimulation

Using a mixed lymphocyte reaction (57), it has been shown that 1–10% of T cell in peripheral blood can be activated (58). As mentioned before, the number of HLA mismatches between donor and recipient is a primary driving force that mobilized a larger fraction of T cells than nominal antigens. Whether alloreactive T cells are activated by the high number of new antigens presented by donor HLA or by the large number of different allo-pHLA complexes (or both) is still under debate, and the two hypotheses are not mutually exclusives. The indirect pathway further enhances the reactivity of recipient T cells toward allogeneic graft. Indeed, peptides presented by MHC molecules derived predominantly from MHC-related molecules (59–61). The introduction of donor HLA molecules will thus lead to the introduction of great pool of new peptides that can mobilized a large fraction of recipient T cells.

It is now also well accepted that memory T cells generated prior transplantation constitute a major hurdle for long-term graft acceptance. Chronic viruses such as EBV and CMV induce the generation of a large pool of memory T cells. For instance, 10% of both the CD4 and CD8 memory compartments in blood are reactive to HCMV (62). The cross-reactivity between virus-specific T cells and allogeneic HLA has been extensively documented (63). EBV or CMV specific CD8 T cells exhibit frequently a cross-reactivity toward allogeneic MHC class I complexes (64–68). Similar observations have been reported for CD4 T cells specific for EBV or CMV (69–71). Virus-specific T cells that cross-react with alloantigens have been shown in experimental models to proliferate in response to a transplanted allograft *in vivo* (72). For instance, LCMV-specific CD8 T cells generated after infection of mice with Armstrong strain of LCMV are able to vigorously proliferate *in vivo* after skin transplantation and ultimately to mediate skin graft rejection (72).

### Tracking Anti-Donor Response by the Investigation of TCR Vβ Repertoire: From Low Resolution Technique to High Throughput Sequencing

**Figure 2:**
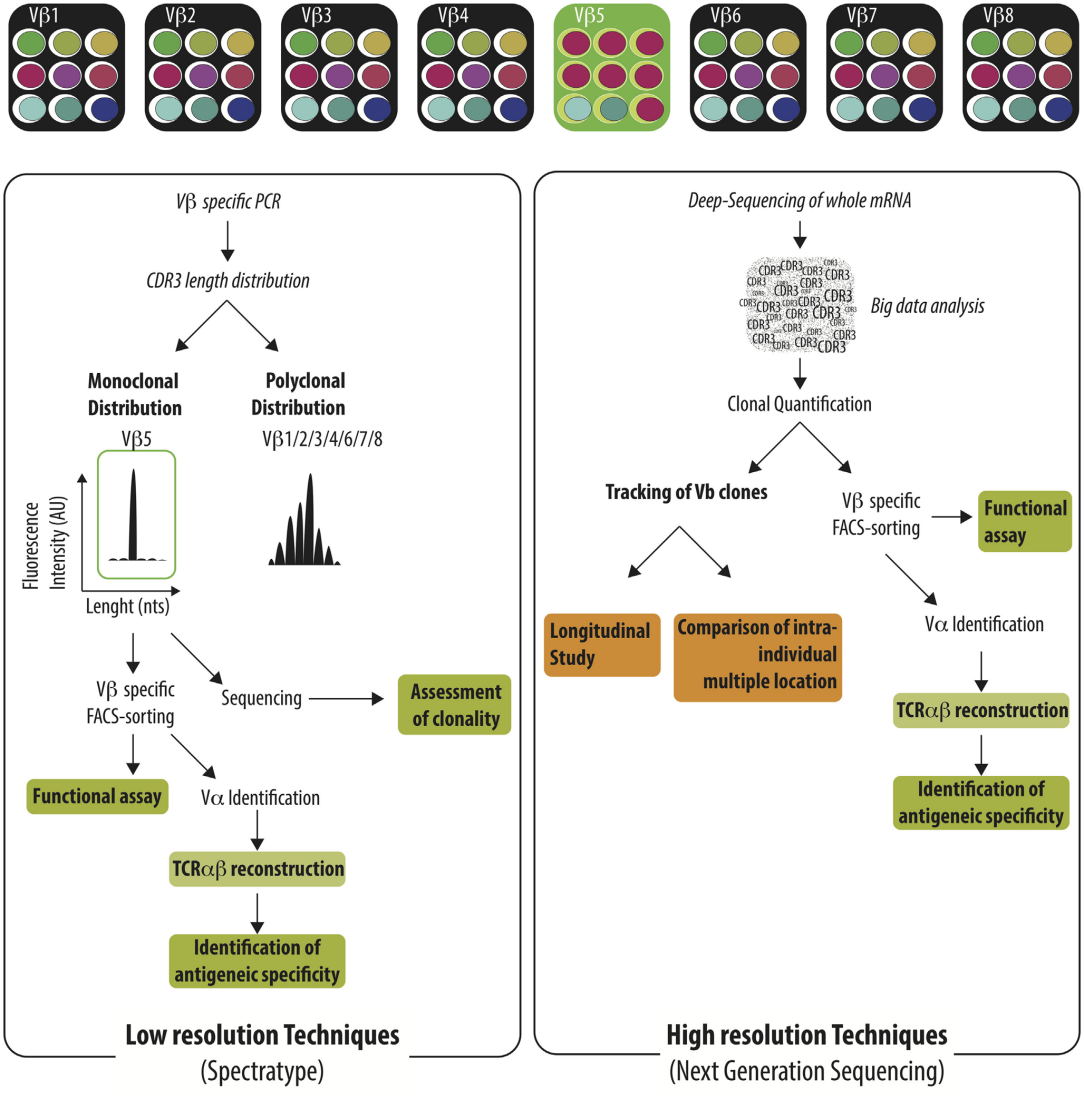
**Characterization of the TCR Vβ repertoire by low resolution and high resolution technique**. Immune challenge leads to the selection of T cells harboring specific TCRαβ among the highly diverse TCRαβ repertoire. Antigen-specific T cells could be identified by low-resolution techniques (e.g., spectratype) or high-resolution techniques (e.g., NGS). Low-resolution techniques are aiming to identify Vβ families that exhibit monoclonal distribution of their CDR3 length distribution using Vβ specific PCR and spectratyping. The clonality of the identified Vβ families needs to be confirmed by the sequencing of the PCR product. Vβ-specific T cell purification enables later to perform functional assay or to reconstruct the TCRαβ in order to identify the recognized antigen. Deep-sequencing of TCR Vβ region identify the sequence of each TCR Vβ and intensive bio-informatic process is needed to quantify the abundance of the different T cell clones. Given the burden of data generated, the Next-Generation Sequencing is well-fitted to track T cell clones in time or across different anatomic sites.

Given the size of anti-donor T cell pool, great efforts have been paid to track the immune-response using the analysis of TCR Vβ repertoire and to correlate specific usage of TCR Vβ repertoire with graft status or graft outcome. Before presenting the available reports, it is necessary to present the two major methods used to investigate TCR Vβ usage; a low resolution (spectratype alone or TcLandscape when combined with quantitative analysis) and, more recently, a high resolution (deep-sequencing of TCR Vβ region) approach (Figure 2). The low-resolution technique is based on the analysis of the length of the CDR3 region whereas the high-resolution technique identifies the sequence of each TCR Vβ and later quantifies the abundance of the different T cell clones.

Each TCR Vβ family is composed of T cells with various lengths of their CDR3 region. The distribution of the CDR3 length can be assessed by spectratype (73, 74). A broad spectrum of profiles can be identified ranging from a Gaussian-like profile to a highly restricted profile, highlighting the absence of selection of T cell, or the expansion of T cell clones, respectively. Different analytic tools have been used to characterize the CDR3 length distribution (75–78). The qualitative assessment of the TCR Vβ repertoire can be complemented by the quantification of the different Vβ families at the mRNA level using qRT-PCR (79–81) or at the cellular level using flow-cytometry (82). Such techniques still offer several benefits over higher resolution techniques such as their cost, the short time frame to obtain results, and the generation of a reasonable amount of data can be also displayed as “visible” pattern as an “X-ray” of the global TCR alteration in a specific pathological context (83–86). A rapid survey of the usage of the TCR Vβ repertoire can be efficiently performed, guiding further investigations focused on targeted TCR Vβ families. At the other range of the resolution spectrum, deep-sequencing of TCR Vβ obtains a full picture of the usage of T cell repertoire with deep or ultra-deep resolution. The availability of all TCR Vβ sequences allows for the precise appraisal of the distribution of the different T cell clones especially across different biological compartments (76). Furthermore, with a complete TCR Vβ sequencing, researchers can investigate the similarity of T cell sequences between biological compartments or individuals and take advantage of public repository databases to assess the specificity of a sequence and potentially to reconstruct the TCR in order to search for the recognized peptides. However, the amount of data generated using this technique is extremely high and efficient bio-informatics tools specifically devoted to the analysis are needed to identify meaningful information in the ocean of data. The accessibility of deep-sequencing is likely to be broaden in the near future thanks to the advances in bio-informatics tools and the reduction of the cost.

Low-resolution techniques have been used to investigate the usage of TCR Vβ repertoire in kidney transplant recipients with various clinical outcomes or at various time points posttransplantation (86–88). Using the combination of spectratyping and quantitative assessment of the TCR Vβ transcript, we have been able to define direct or indirect allorecognition patterns in an experiment model of allograft in congenic rats (52, 79, 80). Using the same approach, we reported that patients with biopsy-proven chronic antibody-mediated (CAMR) rejection exhibits strong alterations of their TCR Vβ repertoire correlating with the level of graft lesions classified with Banff classification (87). In contrast, operationally tolerant patients [i.e., patients off-immunosuppression for more than 12 months with a well-functioning graft (89–91)] exhibit a polyclonal TCR Vβ repertoire (87). A large cohort of patients with stable graft function for more than 5 years post-transplantation had been prospectively recruited in our center with stringent clinical and demographic inclusion criteria in order to obtain a homogeneous population. Nevertheless, we could highlight that the usage of TCR Vβ repertoire is highly heterogeneous ranging from the absence of clonal selection (similar to operational tolerance) to an accumulation of selected T cells (as for CAMR rejection) (87). The presence of altered TCR Vβ repertoire has been previously reported in a rat model of CAMR (92) in which similar CD8 clones could be identified in the blood and in the graft (93). In a large prospective study of kidney transplant recipients with a stable graft function for more than 5 years, we show that the altered TCR Vβ repertoire was due to an accumulation of TEMRA (T cell Effector Memory re-expressing CD45RA; CD45RA^+^CCR7^−^) CD8 T cells with an activated profile (CD27^−^CD28^−^), a high expression of cytotoxic molecules, perforin (PERF) and Granzym B (GZM-B), T-bet, and CD57 and the ability to secrete TNF-α and IFN-γ (88). Of interest, stable patients who have an increase in differentiated TEMRA CD8 T cells have a twofold higher risk of long-term graft dysfunction (88). Of note, using a similar strategy, Kim et al. recently reported that clonal CD8 T cell could be evidenced in human transplanted hand, with several TCR clonal selections persisting at least 100 days (among the 178 days of surveillance) (94). Collectively, these data highlight that a low-resolution technic provides key features as for the accumulation of selected T cell clones that can be used to monitor the kidney transplant recipients.

A major drawback of spectratype-based method is its intrinsic low resolution as multiple T cell clones could share the same CDR3 length. It is necessary to sequence the TCR Vβ chain with an altered CDR3 length distribution to assess the clonality of a given Vβ family. However, we recently compared spectratype or next generation sequencing (NGS) techniques to characterize the TCR Vβ repertoire in the blood, the cerebral spinal fluid (CSF), and the central nervous system (CNS) of patients with multiple sclerosis (76). Both methods were as efficient to highlight the similarity of TCR Vβ repertoire between CSF and CNS (≈80% of TCR Vβ clones identified in the CNS were also found in the CSF) and to identify ≈50% of the TCR Vβ clones using blood CD8 sample (76).

As previously discussed, the size of donor-reactive T cell repertoire is large and constitutes a limitation to the use of deep-sequencing approach. It may thus be a naive approach to perform NGS on unfractioned T cells with the aim to identify T cell clones specific to a given situation, such as kidney transplantation or viral infection; a two-step approach is needed. The first step is to purify the T cell population of interest based on the expression of phenotypic (using tetramer for instance) or functional (e.g., cytokine secretion, proliferation) markers. The in-depth characterization of TCR Vβ of T cell population of interest allows for the definition of a signature that can be later used as a tag when unpurified samples are analyzed. This approach has been used to track CMV- or BK-specific T cell clones (95) or alloreactive T cells (96, 97). The first report hypothesizing such an approach in the transplant context has been published by the group of Leventhal (96). Using healthy volunteers, this study aimed to assess breadth, clonal structure, and dynamics of the alloreactive T cell repertoire. After 7 days of MLR, the proliferating T cells were purified according to the dilution of cell division dye. By comparing the number of clones before culture and in the proliferated MLR responder, two types of alloreactive clones were identified, low- (i.e., unobserved in pre-culture sample and ≥10 T cells after MLR) and high-abundance pre-culture clones (i.e., present in pre-culture sample and ≥10× enriched after MLR). More than 11,000 low-abundant clones and more than 2,000 high-abundant clones were detected in the different experiments. These data provide new evidences of the large size of the alloreactive T cell pool.

This approach was used recently to track donor-reactive T cells in kidney transplant recipients (97). The fingerprint of donor-reactive T cell repertoire was established before transplantation by deep-sequencing of proliferating CD4 and CD8 T cells after 6 days of MLR. The fingerprint of donor-reactive T cells was monitored later after transplantation without the need to perform MLR. The team of Sykes provides evidences that tolerance induction protocol based on combined kidney and non-myeloablative bone marrow transplantation results in a reduction of donor-alloreactive T cell clones. However, this decrease was neither observed in the patient that failed to respond to the tolerant inducing protocol nor in patients with standard immunosuppressive regimens. Pre-transplant identification of donor-reactive T cell clones before transplantation could thus be a means to track the activation of the immune system by allogeneic graft. The studies of Emerson (96) and Morris (97) showed that the anti-donor fingerprint is stable over-time in healthy volunteers. Given the design of the assay, only pre-existing clones could be tracked. It would be of great value to compare the anti-donor clone repertoire before and after transplantation, starting each time from a direct MLR assay to investigate if new anti-donor T cells arise after transplantation. Indeed, infections that occurred frequently after transplantation could generate virus-specific T cells with an allogeneic cross-reactivity potential (71). Moreover, not all proliferating cells after 6 days of MLR are *per se* donor-specific as proliferation of T cells could also be linked to bystander stimulation (98).

## Concluding Remarks

Will transplant immunologists be able to track the rise and the expansion of donor-specific T cells and would this approach be widely available and useful to the clinical management are still open questions. High-through put techniques that have recently emerged are certainly an important step forward. Nevertheless, the high cross-reactivity of T cells is a major hurdle to identify the trigger of the expansion of donor-reactive T cells, as donor antigen, viral peptides, and other environmental antigens can lead to the selection of donor-specific T cells. While promising, the study of TCR alteration has not overcome the double difficulties of offering an accessible technical presentation of the data and a validated correlation with clinical outcomes. Therefore, longitudinal studies to test the reactivity of recipient T cells against donor antigens at different time points are needed.

## Author Contributions

ND, SB, and J-P S wrote the review.

## Conflict of Interest Statement

The authors declare that the research was conducted in the absence of any commercial or financial relationships that could be construed as a potential conflict of interest.

## Abbreviations

CAMR, chronic antibody-mediated; CDR, cluster differentiation region; CMV, cytomegalovirus; CNS, central nervous system; CSF, cerebral spinal fluid; EBV, Epstein–Barr virus; GZMb, Granzym B; HLA, human leukocyte antigen; IFNg, interferon g; ITAM, immunoreceptor tyrosine activation motifs; MHC, major histocompatibility complex; MLR, mixed lymphocyte reaction; NGS, next generation sequencing; PERF, perforin; TCR, T cell repertoire; TEMRA, T cell effector memory re-expressing CD45RA; TNFa, Tumor necrosis factor.

## References

[1] Tonegawa S. Somatic generation of antibody diversity. Nature (1983) 302:575–81. 10.1038/302575a0 6300689

[2] Rowen L Koop BF Hood L. The complete 685-kilobase DNA sequence of the human beta T cell receptor locus. Science (1996) 272:1755–62. 10.1126/science.272.5269.1755 8650574

[3] Boysen C Simon MI Hood L. Analysis of the 1.1-Mb human alpha/delta T-cell receptor locus with bacterial artificial chromosome clones. Genome Res (1997) 7:330–8. 9110172 10.1101/gr.7.4.330

[4] Zarnitsyna VI Evavold BD Schoettle LN Blattman JN Antia R. Estimating the diversity, completeness, and cross-reactivity of the T cell repertoire. Front Immunol (2013) 4:485. 10.3389/fimmu.2013.00485 24421780 PMC3872652

[5] Quiròs Roldàn E Sottini A Bettinardi A Albertini A Imberti L Primi D. Different TCRBV genes generate biased patterns of V-D-J diversity in human T cells. Immunogenetics (1995) 41:91–100. 7806301 10.1007/BF00182318

[6] Warren RL Freeman JD Zeng T Choe G Munro S Moore R Exhaustive T-cell repertoire sequencing of human peripheral blood samples reveals signatures of antigen selection and a directly measured repertoire size of at least 1 million clonotypes. Genome Res (2011) 21:790–7. 10.1101/gr.115428.110 21349924 PMC3083096

[7] Lafaille JJ DeCloux A Bonneville M Takagaki Y Tonegawa S. Junctional sequences of T cell receptor gamma delta genes: implications for gamma delta T cell lineages and for a novel intermediate of V-(D)-J joining. Cell (1989) 59:859–70. 10.1016/0092-8674(89)90609-0 2590942

[8] Cabaniols JP Fazilleau N Casrouge A Kourilsky P Kanellopoulos JM. Most alpha/beta T cell receptor diversity is due to terminal deoxynucleotidyl transferase. J Exp Med (2001) 194:1385–90. 10.1084/jem.194.9.1385 11696602 PMC2195970

[9] Murugan A Mora T Walczak AM Callan CG. Statistical inference of the generation probability of T-cell receptors from sequence repertoires. Proc Natl Acad Sci U S A (2012) 109:16161–6. 10.1073/pnas.1212755109 22988065 PMC3479580

[10] Tallquist MD Weaver AJ Pease LR. Degenerate recognition of alloantigenic peptides on a positive-selecting class I molecule. J Immunol (1998) 160:802–9. 9551915

[11] Udaka K Wiesmüller KH Kienle S Jung G Walden P. Self-MHC-restricted peptides recognized by an alloreactive T lymphocyte clone. J Immunol (1996) 157:670–8. 8752916

[12] Garcia KC Degano M Stanfield RL Brunmark A Jackson MR Peterson PA An alpha beta T cell receptor structure at 2.5 A and its orientation in the TCR-MHC complex. Science (1996) 274:209–19. 10.1126/science.274.5285.209 8824178

[13] Rossjohn J Gras S Miles JJ Turner SJ Godfrey DI McCluskey J T cell antigen receptor recognition of antigen-presenting molecules. Annu Rev Immunol (2015) 33:169–200. 10.1146/annurev-immunol-032414-112334 25493333

[14] Garcia KC. Reconciling views on T cell receptor germline bias for MHC. Trends Immunol (2012) 33:429–36. 10.1016/j.it.2012.05.005 22771140 PMC3983780

[15] Kappler JW Wade T White J Kushnir E Blackman M Bill J A T cell receptor V beta segment that imparts reactivity to a class II major histocompatibility complex product. Cell (1987) 49:263–71. 10.1016/0092-8674(87)90567-8 3471350

[16] Beringer DX Kleijwegt FS Wiede F van der Slik AR Loh KL Petersen J T cell receptor reversed polarity recognition of a self-antigen major histocompatibility complex. Nat Immunol (2015) 16:1153–61. 10.1038/ni.3271 26437244

[17] Matzinger P Bevan MJ Hypothesis: why do so many lymphocytes respond to major histocompatibility antigens? Cell Immunol (1977) 29:1–5. 10.1016/0008-8749(77)90269-6 300293

[18] Owens T Liddell ME Crispe IN. Derivation from an alloreactive T-cell line of a clone which cross-reacts with a self H2-E-restricted minor alloantigen. Cell Immunol (1984) 85:436–46. 10.1016/0008-8749(84)90257-0 6609002

[19] Birnbaum ME Mendoza JL Sethi DK Dong S Glanville J Dobbins J Deconstructing the peptide-MHC specificity of T cell recognition. Cell (2014) 157:1073–87. 10.1016/j.cell.2014.03.047 24855945 PMC4071348

[20] Jerne NK The natural-selection theory of antibody formation. Proc Natl Acad Sci U S A (1955) 41:849–57. 10.1073/pnas.41.11.849 16589759 PMC534292

[21] Jerne NK The somatic generation of immune recognition. Eur J Immunol (1971) 1:1–9. 10.1002/eji.1830010102 14978855

[22] Mason D A very high level of crossreactivity is an essential feature of the T-cell receptor. Immunol Today (1998) 19:395–404. 10.1016/S0167-5699(98)01299-7 9745202

[23] Sewell AK. Why must T cells be cross-reactive? Nat Rev Immunol (2012) 12:669–77. 10.1038/nri3279 22918468 PMC7097784

[24] Arstila TP A direct estimate of the human T cell receptor diversity. Science (1999) 286:958–61. 10.1126/science.286.5441.958 10542151

[25] Robins HS Campregher PV Srivastava SK Wacher A Turtle CJ Kahsai O Comprehensive assessment of T-cell receptor beta-chain diversity in alphabeta T cells. Blood (2009) 114:4099–107. 10.1182/blood-2009-04-217604 19706884 PMC2774550

[26] Rudd BD Venturi V Li G Samadder P Ertelt JM Way SS Nonrandom attrition of the naive CD8+ T-cell pool with aging governed by T-cell receptor:pMHC interactions. Proc Natl Acad Sci USA (2011) 108:13694–9. 10.1073/pnas.1107594108 21813761 PMC3158207

[27] Akue AD Lee J-Y Jameson SC. Derivation and maintenance of virtual memory CD8 T cells. J Immunol (2012) 188:2516–23. 10.4049/jimmunol.1102213 22308307 PMC3294185

[28] Haluszczak C Akue AD Hamilton SE Johnson LDS Pujanauski L Teodorovic L The antigen-specific CD8+ T cell repertoire in unimmunized mice includes memory phenotype cells bearing markers of homeostatic expansion. J Exp Med (2009) 206:435–48. 10.1084/jem.20081829 19188498 PMC2646575

[29] Rudd BD Venturi V Smithey MJ Way SS Davenport MP Nikolich-Žugich J. Diversity of the CD8+ T cell repertoire elicited against an immunodominant epitope does not depend on the context of infection. J Immunol (2010) 184:2958–65. 10.4049/jimmunol.0903493 20164421 PMC4161216

[30] Su LF Kidd BA Han A Kotzin JJ Davis MM Virus-specific CD4+ memory-phenotype T cells are abundant in unexposed adults. Immunity (2013) 38:373–83. 10.1016/j.immuni.2012.10.021 23395677 PMC3626102

[31] Sprent CDSJ Homeostasis of naive and memory T cells. Immunity (2008) 29:848–62. 10.1016/j.immuni.2008.11.002 19100699

[32] Yu W Jiang N Ebert PJR Kidd BA Müller S Lund PJ Clonal deletion prunes but does not eliminate self-specific αβ CD8(+) T lymphocytes. Immunity (2015) 42:929–41. 10.1016/j.immuni.2015.05.001 25992863 PMC4455602

[33] Brodin P Jojic V Gao T Bhattacharya S Angel CJL Furman D Variation in the human immune system is largely driven by non-heritable influences. Cell (2015) 160:37–47. 10.1016/j.cell.2014.12.020 25594173 PMC4302727

[34] Stewart AJ Devlin PM. The history of the smallpox vaccine. J Infect (2006) 52:329–34. 10.1016/j.jinf.2005.07.021 16176833

[35] Welsh RM Che JW Brehm MA Selin LK. Heterologous immunity between viruses. Immunol Rev (2010) 235:244–66. 10.1111/j.0105-2896.2010.00897.x 20536568 PMC2917921

[36] Selin LK Varga SM Wong IC Welsh RM. Protective heterologous antiviral immunity and enhanced immunopathogenesis mediated by memory T cell populations. J Exp Med (1998) 188:1705–15. 10.1084/jem.188.9.1705 9802982 PMC2212518

[37] Mathurin KS Martens GW Kornfeld H Welsh RM. CD4 T-cell-mediated heterologous immunity between mycobacteria and poxviruses. J Virol (2009) 83:3528–39. 10.1128/JVI.02393-08 19193795 PMC2663272

[38] Chen HD Fraire AE Joris I Welsh RM Selin LK. Specific history of heterologous virus infections determines anti-viral immunity and immunopathology in the lung. Am J Pathol (2003) 163:1341–55. 10.1016/S0002-9440(10)63493-1 14507643 PMC1868309

[39] Nilges K Höhn H Pilch H Neukirch C Freitag K Talbot PJ Human papillomavirus type 16 E7 peptide-directed CD8+ T cells from patients with cervical cancer are cross-reactive with the coronavirus NS2 protein. J Virol (2003) 77:5464–74. 10.1128/JVI.77.9.5464-5474.2003 12692247 PMC153943

[40] Clute SC Naumov YN Watkin LB Aslan N Sullivan JL Thorley-Lawson DA Broad cross-reactive TCR repertoires recognizing dissimilar Epstein-Barr and influenza A virus epitopes. J Immunol (2010) 185:6753–64. 10.4049/jimmunol.1000812 21048112 PMC3738202

[41] Billington WD Influence of immunological dissimilarity of mother and foetus on size of placenta in mice. Nature (1964) 202:317–8. 10.1038/202317a0 14167813

[42] Ober C Hyslop T Elias S Weitkamp LR Hauck WW. Human leukocyte antigen matching and fetal loss: results of a 10 year prospective study. Hum Reprod (1998) 13:33–8. 10.1093/humrep/13.1.33 9512225

[43] Kappler JW Roehm N Marrack P T cell tolerance by clonal elimination in the thymus. Cell (1987) 49:273–80. 10.1016/0092-8674(87)90568-X 3494522

[44] Luz JG Huang M Garcia KC Rudolph MG Apostolopoulos V Teyton L Structural comparison of allogeneic and syngeneic T cell receptor-peptide-major histocompatibility complex complexes: a buried alloreactive mutation subtly alters peptide presentation substantially increasing V(beta) Interactions. J Exp Med (2002) 195:1175–86. 10.1084/jem.20011644 11994422 PMC2193710

[45] Reiser JB Darnault C Guimezanes A Grégoire C Mosser T Schmitt-Verhulst AM Crystal structure of a T cell receptor bound to an allogeneic MHC molecule. Nat Immunol (2000) 1:291–7. 10.1038/79728 11017099

[46] Doxiadis II Smits JM Schreuder GM Persijn GG van Houwelingen HC van Rood JJ Association between specific HLA combinations and probability of kidney allograft loss: the taboo concept. Lancet (1996) 348:850–3. 10.1016/S0140-6736(96)02296-9 8826810

[47] Fleischhauer K Kernan NA O’Reilly RJ Dupont B Yang SY Bone marrow-allograft rejection by T lymphocytes recognizing a single amino acid difference in HLA-B44. N Engl J Med (1990) 323:1818–22. 10.1056/NEJM199012273232607 2247120

[48] Keever CA Leong N Cunningham I Copelan EA Avalos BR Klein J HLA-B44-directed cytotoxic T cells associated with acute graft-versus-host disease following unrelated bone marrow transplantation. Bone Marrow Transplant (1994) 14:137–45. 7951101

[49] Fleischhauer K Avila D Vilbois F Traversari C Bordignon C Wallny HJ. Characterization of natural peptide ligands for HLA-B*4402 and -B*4403: implications for peptide involvement in allorecognition of a single amino acid change in the HLA-B44 heavy chain. Tissue Antigens (1994) 44:311–7. 10.1111/j.1399-0039.1994.tb02401.x 7878657

[50] Macdonald WA Purcell AW Mifsud NA Ely LK Williams DS Chang L A naturally selected dimorphism within the HLA-B44 supertype alters class I structure, peptide repertoire, and T cell recognition. J Exp Med (2003) 198:679–91. 10.1084/jem.20030066 12939341 PMC2194191

[51] Covassin L Laning J Abdi R Langevin DL Phillips NE Shultz LD Human peripheral blood CD4 T cell-engrafted non-obese diabetic-scid IL2rγ(null) H2-Ab1 (tm1Gru) Tg (human leucocyte antigen D-related 4) mice: a mouse model of human allogeneic graft-versus-host disease. Clin Exp Immunol (2011) 166:269–80. 10.1111/j.1365-2249.2011.04462.x 21985373 PMC3219902

[52] Guillet M Gagne K Lair D Heslan J-M Dore J-C Soulillou J-P Different patterns of TCR beta chain regulation following allo- and xeno-transplantation. Xenotransplantation (2004) 11:315–22. 10.1111/j.1399-3089.2004.00136.x 15196125

[53] Schilham MW Lang R Benner R Zinkernagel RM Hengartner H. Characterization of an Lyt-2+ alloreactive cytotoxic T cell clone specific for H-2Db that cross-reacts with I-Ek. J Immunol (1986) 137:2748–54. 3489776

[54] Logunova NN Viret C Pobezinsky LA Miller SA Kazansky DB Sundberg JP Restricted MHC-peptide repertoire predisposes to autoimmunity. J Exp Med (2005) 202:73–84. 10.1084/jem.20050198 15998789 PMC2212910

[55] Huseby ES White J Crawford F Vass T Becker D Pinilla C How the T cell repertoire becomes peptide and MHC specific. Cell (2005) 122:247–60. 10.1016/j.cell.2005.05.013 16051149

[56] Yin L Huseby E Scott-Browne J Rubtsova K Pinilla C Crawford F A single T cell receptor bound to major histocompatibility complex class I and class II glycoproteins reveals switchable TCR conformers. Immunity (2011) 35:23–33. 10.1016/j.immuni.2011.04.017 21683626 PMC3160269

[57] Albertini RJ Bach FH. Quantitative assay of antigenic disparity at hl-a-the major histocompatibility locus in man. J Exp Med (1968) 128:639–51. 10.1084/jem.128.4.639 19867302 PMC2138551

[58] Suchin EJ Langmuir PB Palmer E Sayegh MH Wells AD Turka LA. Quantifying the frequency of alloreactive T cells in vivo: new answers to an old question. J Immunol (2001) 166:973–81. 10.4049/jimmunol.166.2.973 11145675

[59] Chicz RM Urban RG Lane WS Gorga JC Stern LJ Vignali DA Predominant naturally processed peptides bound to HLA-DR1 are derived from MHC-related molecules and are heterogeneous in size. Nature (1992) 358:764–8. 10.1038/358764a0 1380674

[60] Chicz RM Urban RG Gorga JC Vignali DA Lane WS Strominger JL. Specificity and promiscuity among naturally processed peptides bound to HLA-DR alleles. J Exp Med (1993) 178:27–47. 10.1084/jem.178.1.27 8315383 PMC2191090

[61] Rudensky AY Preston-Hurlburt P Hong SC Barlow A Janeway CA. Sequence analysis of peptides bound to MHC class II molecules. Nature (1991) 353:622–7. 10.1038/353622a0 1656276

[62] Sylwester AW Mitchell BL Edgar JB Taormina C Pelte C Ruchti F Broadly targeted human cytomegalovirus-specific CD4+ and CD8+ T cells dominate the memory compartments of exposed subjects. J Exp Med (2005) 202:673–85. 10.1084/jem.20050882 16147978 PMC2212883

[63] Smith C Miles JJ Khanna R. Advances in direct T-cell alloreactivity: function, avidity, biophysics and structure. Am J Transplant (2012) 12:15–26. 10.1111/j.1600-6143.2011.03863.x 22152064

[64] Burrows SR Silins SL Moss DJ Khanna R Misko IS Argaet VP T cell receptor repertoire for a viral epitope in humans is diversified by tolerance to a background major histocompatibility complex antigen. J Exp Med (1995) 182:1703–15. 10.1084/jem.182.6.1703 7500015 PMC2192251

[65] Morice A Charreau B Neveu B Brouard S Soulillou J-P Bonneville M Cross-reactivity of herpesvirus-specific CD8 T cell lines toward allogeneic class I MHC molecules. PLoS One (2010) 5:e12120. 10.1371/journal.pone.0012120 20711433 PMC2920819

[66] Burrows SR Khanna R Burrows JM Moss DJ. An alloresponse in humans is dominated by cytotoxic T lymphocytes (CTL) cross-reactive with a single Epstein-Barr virus CTL epitope: implications for graft-versus-host disease. J Exp Med (1994) 179:1155–61. 10.1084/jem.179.4.1155 7511682 PMC2191444

[67] Burrows SR Silins SL Khanna R Burrows JM Rischmueller M McCluskey J Cross-reactive memory T cells for Epstein-Barr virus augment the alloresponse to common human leukocyte antigens: degenerate recognition of major histocompatibility complex-bound peptide by T cells and its role in alloreactivity. Eur J Immunol (1997) 27:1726–36. 10.1002/eji.1830270720 9247584

[68] Rist M Smith C Bell MJ Burrows SR Khanna R. Cross-recognition of HLA DR4 alloantigen by virus-specific CD8+ T cells: a new paradigm for self-/nonself-recognition. Blood (2009) 114:2244–53. 10.1182/blood-2009-05-222596 19617574

[69] Landais E Morice A Long HM Haigh TA Charreau B Bonneville M EBV-specific CD4+ T cell clones exhibit vigorous allogeneic responses. J Immunol (2006) 177:1427–33. 10.4049/jimmunol.177.3.1427 16849448

[70] Elkington R Khanna R. Cross-recognition of human alloantigen by cytomegalovirus glycoprotein-specific CD4+ cytotoxic T lymphocytes: implications for graft-versus-host disease. Blood (2005) 105:1362–4. 10.1182/blood-2004-07-2602 15459005

[71] Amir AL D’Orsogna LJA Roelen DL van Loenen MM Hagedoorn RS de Boer R Allo-HLA reactivity of virus-specific memory T cells is common. Blood (2010) 115:3146–57. 10.1182/blood-2009-07-234906 20160165

[72] Brehm MA Daniels KA Priyadharshini B Thornley TB Greiner DL Rossini AA Allografts stimulate cross-reactive virus-specific memory CD8 T cells with private specificity. Am J Transplant (2010) 10:1738–48. 10.1111/j.1600-6143.2010.03161.x 20659086 PMC2911646

[73] Gorski J Yassai M Zhu X Kissela B Kissella B corrected to Kissela B Keever C Circulating T cell repertoire complexity in normal individuals and bone marrow recipients analyzed by CDR3 size spectratyping. Correlation with immune status. J Immunol (1994) 152:5109–19. 8176227

[74] Cochet M Pannetier C Regnault A Darche S Leclerc C Kourilsky P. Molecular detection and in vivo analysis of the specific T cell response to a protein antigen. Eur J Immunol (1992) 22:2639–47. 10.1002/eji.1830221025 1327801

[75] Miqueu P Guillet M Degauque N Dore J-C Soulillou J-P Brouard S. Statistical analysis of CDR3 length distributions for the assessment of T and B cell repertoire biases. Mol Immunol (2007) 44:1057–64. 10.1016/j.molimm.2006.06.026 16930714

[76] Salou M Garcia A Michel L Gainche-Salmon A Loussouarn D Nicol B Expanded CD8 T-cell sharing between periphery and CNS in multiple sclerosis. Ann Clin Transl Neurol (2015) 2:609–22. 10.1002/acn3.199 26125037 PMC4479522

[77] Degauque N Boeffard F Foucher Y Ballet C Brouard S Soulillou J-P. The blood of healthy individuals exhibits CD8 T cells with a highly altered TCR Vb repertoire but with an unmodified phenotype. PLoS One (2011) 6:e21240. 10.1371/journal.pone.0021240 21738624 PMC3124488

[78] Brouard S Vanhove B Gagne K Neumann A Douillard P Moreau A T cell repertoire alterations of vascularized xenografts. J Immunol (1999) 162:3367–77. 10092791

[79] Sebille F Gagne K Guillet M Degauque N Pallier A Brouard S Direct recognition of foreign MHC determinants by naive T cells mobilizes specific Vbeta families without skewing of the complementarity-determining region 3 length distribution. J Immunol (2001) 167:3082–8. 10.4049/jimmunol.167.6.3082 11544292

[80] Gagne K Brouard S Giral M Sebille F Moreau A Guillet M Highly altered V beta repertoire of T cells infiltrating long-term rejected kidney allografts. J Immunol (2000) 164:1553–63. 10.4049/jimmunol.164.3.1553 10640774

[81] Thaunat O Graff-Dubois S Brouard S Gautreau C Varthaman A Fabien N Immune responses elicited in tertiary lymphoid tissues display distinctive features. PLoS One (2010) 5:e11398. 10.1371/journal.pone.0011398 20613979 PMC2894881

[82] Pilch H Höhn H Freitag K Neukirch C Necker A Haddad P Improved assessment of T-cell receptor (TCR) VB repertoire in clinical specimens: combination of TCR-CDR3 spectratyping with flow cytometry-based TCR VB frequency analysis. Clin Diagn Lab Immunol (2002) 9:257–66. 10.1128/CDLI.9.2.257-266.2002 11874861 PMC119929

[83] Degauque N Schadendorf D Brouard S Guillet M Sébille F Höhn H Blood T-cell Vbeta transcriptome in melanoma patients. Int J Cancer (2004) 110:721–9. 10.1002/ijc.20149 15146562

[84] Laplaud DA Berthelot L Miqueu P Bourcier K Moynard J Oudinet Y Serial blood T cell repertoire alterations in multiple sclerosis patients; correlation with clinical and MRI parameters. J Neuroimmunol (2006) 177:151–60. 10.1016/j.jneuroim.2006.05.006 16806500

[85] Guillet M Andrieu M Braudeau C Ruiz C Daniel N Pallier A Serial evolution of TCR beta chain transcript mobilization in HIV type-1-infected patients following vaccine immune stimulation and HAART interruption. AIDS Res Hum Retroviruses (2006) 22:648–56. 10.1089/aid.2006.22.648 16831089

[86] Brouard S Dupont A Giral M Louis S Lair D Braudeau C Operationally tolerant and minimally immunosuppressed kidney recipients display strongly altered blood T-cell clonal regulation. Am J Transplant (2005) 5:330–40. 10.1111/j.1600-6143.2004.00700.x 15643993

[87] Miqueu P Degauque N Guillet M Giral M Ruiz C Pallier A Analysis of the peripheral T-cell repertoire in kidney transplant patients. Eur J Immunol (2010) 40:3280–90. 10.1002/eji.201040301 21061447

[88] Yap M Boeffard F Clave E Pallier A Danger R Giral M Expansion of highly differentiated cytotoxic terminally differentiated effector memory CD8+ T cells in a subset of clinically stable kidney transplant recipients: a potential marker for late graft dysfunction. J Am Soc Nephrol (2014) 25:1856–68. 10.1681/ASN.2013080848 24652799 PMC4116064

[89] Brouard S Mansfield E Braud C Li L Giral M Hsieh S-C Identification of a peripheral blood transcriptional biomarker panel associated with operational renal allograft tolerance. Proc Natl Acad Sci U S A (2007) 104:15448–53. 10.1073/pnas.0705834104 17873064 PMC2000539

[90] Brouard S Pallier A Renaudin K Foucher Y Danger R Devys A The natural history of clinical operational tolerance after kidney transplantation through twenty-seven cases. Am J Transplant (2012) 12:3296–307. 10.1111/j.1600-6143.2012.04249.x 22974211

[91] Roussey-Kesler G Giral M Moreau A Subra JF Legendre C Noël C Clinical operational tolerance after kidney transplantation. Am J Transplant (2006) 6:736–46. 10.1111/j.1600-6143.2006.01280.x 16539630

[92] Ballet C Renaudin K Degauque N Mai HL Boëffard F Lair D Indirect CD4+ TH1 response, antidonor antibodies and diffuse C4d graft deposits in long-term recipients conditioned by donor antigens priming. Am J Transplant (2009) 9:697–708. 10.1111/j.1600-6143.2009.02556.x 19344461

[93] Lair D Degauque N Miqueu P Jovanovic V Guillet M Mérieau E Functional compartmentalization following induction of long-term graft survival with pregraft donor-specific transfusion. Am J Transplant (2007) 7:538–49. 10.1111/j.1600-6143.2006.01660.x 17217443

[94] Kim JY Balamurugan A Azari K Hofmann C Ng HL Reed EF Clonal CD8+ T cell persistence and variable gene usage bias in a human transplanted hand. PLoS One (2015) 10:e0136235 10.1371/journal.pone.0136235 26287728 PMC4546120

[95] Dziubianau M Hecht J Kuchenbecker L Sattler A Stervbo U Rödelsperger C TCR repertoire analysis by next generation sequencing allows complex differential diagnosis of T cell-related pathology. Am J Transplant (2013) 13:2842–54. 10.1111/ajt.12431 24020931

[96] Emerson RO Mathew JM Konieczna IM Robins HS Leventhal JR. Defining the alloreactive T cell repertoire using high-throughput sequencing of mixed lymphocyte reaction culture. PLoS One (2014) 9:e111943. 10.1371/journal.pone.0111943 25365040 PMC4218856

[97] Morris H DeWolf S Robins H Sprangers B LoCascio SA Shonts BA Tracking donor-reactive T cells: evidence for clonal deletion in tolerant kidney transplant patients. Sci Transl Med (2015) 7:ra10–272. 10.1126/scitranslmed.3010760 25632034 PMC4360892

[98] Matesic D Lehmann PV Heeger PS. High-resolution characterization of cytokine-producing alloreactivity in naive and allograft-primed mice. Transplantation (1998) 65:906–14. 10.1097/00007890-199804150-00008 9565093

